# Supplementary material for: Health-related quality of life and associated factors among prisoners in Gondar city prison, Northwest Ethiopia: Using structural equation modeling
Source: PLoS One. 2023 Aug 25;18(8):e0290626. doi: 10.1371/journal.pone.0290626 (PMC10456194; doi:10.1371/journal.pone.0290626)
Supplement: S1 Table — (DOCX) [file pone.0290626.s001.docx]

Table 5: Direct, indirect and total effects of socio-demographic, Health and imprisonment related factors on the four domains of WHOQoL and on global HRQoL among prisoners at Gondar town prison, Northwest Ethiopia, 2022.

| **Characteristics** | **Direct effect**  **(95% CI)** | **Indirect effect**  **(95% CI)** | **Total effect**  **(95% CI)** |
| --- | --- | --- | --- |
| **DV: Physical health** |  |  |  |
| **Age** | -0.286 (-0.362, -0.227) |  |  |
| **Marital status** |  |  |  |
| **Single** |  | 0 |  |
| **Married** |  | -0.025 (-0.049, -0.005) |  |
| **Comorbidity** |  |  |  |
| **No** | 0 | 0 | 0 |
| **Yes** | -0.510 (-0.571, -0.445) | -0.080 (-0.105, -0.058) | -0.590 (-0.651, -0.531) |
| **Work in prison** |  |  |  |
| **No** | 0 |  |  |
| **Yes** | 0.122 (0.078, 0.163) | 0.034 (0.013, 0.058) | 0.156 (0.107, 0.202) |
| **Duration of imprisonment** |  | -0.048 (-0.070, -0.029) |  |
| **Depression** |  |  |  |
| **No** | 0 |  |  |
| **Yes** | 0.122 (0.078, 0.163) |  |  |
|  |  |  |  |
| **DV: Psychological health** |  |  |  |
| **Marital status** |  |  |  |
| **Single** |  | 0 |  |
| **Married** |  | -0.040 (-0.076, -0.006) |  |
| **Comorbidity** |  |  |  |
| **No** | 0 | 0 | 0 |
| **Yes** | -0.220 (-0.289, -0.152) | -0.127 (-0.160, -0.094) | -0.348 (-0.420, -0.271) |
| **Duration of imprisonment** |  | -0.077 (-0.109, -0.047) |  |
| **Work in prison** |  |  |  |
| **No** | 0 | 0 | 0 |
| **Yes** | 0.116 (0.063, 0.165) | 0.054 (0.022, 0.091) | 0.170 (0.112, 0.242) |
| **Visit in prison** |  |  |  |
| **No** |  | 0 |  |
| **Yes** |  | 0.082 (0.048, 0.115) |  |
| **Depression** |  |  |  |
| **No** | 0 |  |  |
| **Yes** | -0.839 (-0.943, -0.743) |  |  |
|  |  |  |  |
| **DV: Social-relationships** |  |  |  |
| **Marital status** |  |  |  |
| **Single** |  | 0 |  |
| **Married** |  | -0.029 (-0.055, -0.005) |  |
| **Comorbidity** |  |  |  |
| **No** | 0 | 0 | 0 |
| **Yes** | -0.165 (-0.250, -0.077) | -0.091 (-0.119, -0.063) | -0.256 (-0.343, -0.168) |
| **Duration of imprisonment** | 0.163 (0.091, 0.248) | -0.055 (-0.080, -0.032) | 0.109 (0.037, 0.194) |
| **Work in prison** |  |  |  |
| **No** |  | 0 |  |
| **Yes** |  | 0.038 (0.015, 0.069) |  |
| **Visit in prison** |  |  |  |
| **No** |  | 0 |  |
| **Yes** |  | 0.059 (0.034, 0.089) |  |
| **Depression** |  |  |  |
| **No** | 0 |  |  |
| **Yes** | -0.440 (-0.528, -0.371) |  |  |
|  |  |  |  |
| **DV: Environmental health** |  |  |  |
| **Age** | 0.094 (0.037, 0.147) |  |  |
| **Marital status** | 0 |  |  |
| **Single** |  | 0 |  |
| **Married** |  | -0.033 (-0.060, -0.006) |  |
| **Comorbidity** |  |  |  |
| **No** | 0 | 0 | 0 |
| **Yes** | -0.163 (-0.218, -0.105) | -0.104 (-0.131, -0.077) | -0.267 (-0.323, -0.204) |
| **Duration of imprisonment** |  | -0.062 (-0.093, -0.039) |  |
| **Work in prison** |  |  |  |
| **No** |  | 0 |  |
| **Yes** |  | 0.044 (0.017, 0.074) |  |
| **Visit in prison** |  |  |  |
| **No** |  | 0 |  |
| **Yes** |  | 0.067 (0.038, 0.095) |  |
| **Depression** |  |  |  |
| **No** | 0 |  |  |
| **Yes** | -0.503 (-0.554, -0.459) |  |  |
|  |  |  |  |
| **DV: Depression** |  |  |  |
| **Marital status** |  |  |  |
| **Single** | 0 |  |  |
| **Married** | 0.065 (0.010, 0.122) |  |  |
| **Comorbidity** |  |  |  |
| **No** | 0 |  |  |
| **Yes** | 0.206 (0.154, 0.260) |  |  |
| **Duration of imprisonment** | 0.124 (0.076, 0.175) |  |  |
| **Work in prison** |  |  |  |
| **No** | 0 |  |  |
| **Yes** | -0.087 (-0.147, -0.031) |  |  |
| **Visit in prison** |  |  |  |
| **No** | 0 |  |  |
| **Yes** | -0.133 (-0.182, -0.076) |  |  |
|  |  |  |  |
| **DV: HRQoL** |  |  |  |
| **Physical health** | 0.534 (0.206, 0.929) |  |  |
| **Environmental health** | 0.467 (0.171, 0.739) |  |  |
| **Age** |  | -0.115 (-0.241, -0.008) |  |
| **Comorbidity** |  |  |  |
| **No** | 0 | 0 | 0 |
| **Yes** |  |  |  |
| **Duration of imprisonment** |  | -0.079 (-0.128, -0.042) |  |
| **Work in prison** |  |  |  |
| **No** |  | 0 |  |
| **Yes** |  | 0.119 (0.072, 0.194) |  |
| **Visit in prison** |  |  |  |
| **No** | 0 | 0 | 0 |
| **Yes** | 0.092 (0.034, 0.144) | 0.059 (0.035, 0.099) | 0.152 (0.097, 0.200) |
| **Depression** |  |  |  |
| **No** |  | 0 |  |
| **Yes** |  | -0.590 (-0.651, -0.531) |  |

Note: *DV= dependent variable, **p<0.05*
