# Supplementary material for: Health-related quality of life and associated factors among prisoners in Gondar city prison, Northwest Ethiopia: Using structural equation modeling
Source: PLoS One. 2023 Aug 25;18(8):e0290626. doi: 10.1371/journal.pone.0290626 (PMC10456194; doi:10.1371/journal.pone.0290626)
Supplement: Attachment — Submitted filename: comments.docx [file pone.0290626.s002.docx]

Thank you for the opportunity to review the manuscript “Health-related quality of life and associated factors among prisoners in Gondar city prison, Northwest Ethiopia: using Structural Equation Modeling”.

**1: Abstract**

1. the authors stated that “General fit indices of the final model were acceptable (x2/df = 1.756, *p* < 0.001, RMSEA = 0.058, TLI = 0.900, CFI = 0.910, and SRMR = 0.057)”. However there were not found these results in the method or result section of the manuscript. The authors supposed to report clearly these findings in the main parts of the paper.

2 .Result: the authors stated that “The mean ± standard deviation score of the overall HRQoL was 53.25±15.12”. I mean, what is the ± sign indicates that? And what is the significance of writing the standard deviation using ±?

3. In abstract, it is better to write in full word i.e., remove aberrations in your abstract section. And in the result and conclusion part of the abstract section almost the same, so in conclusion part please write to answer my question “What's implication for practice based on your results”.

**2: Introduction**

1.During the part of introduction, 79 references were cited, which is very rare. Some of the contents are not relevant to the object of the study, and some cited literatures are wrongly expressed, such as reference 23, 29, 38, 53 and 64. For those reports, the selected population is not focused on prisoners or quality of life.

2. The author stated “Ethiopia’s prison population rate has increased from 94 to 124 per

62 100,000 of the total population between 2000-2011 (4–6).” The data was very old and not updated. The author should be used the updated information to draw the right conclusion.

3. The author also stated that “In Ethiopia, the prevalence of depression, which has a direct negative impact on HRQoL among prisoners was 83.4% in prisons of north-west Ethiopia ……”. Some number written over there seems unreliable, so the authors should write carefully such sensitive numbers.

2. In Hypothesized causal model, what you mean by casual model? Based on the given information on the manuscript, all results was from cross sectional study. So how did you do casual model based on the observational model? Furthermore, there is no reference cited on the hypothetical model(where you develop these hypothetical model). These hypothesized model should be aso presented in your method part of the manuscript.

**3: methods**

1. The authors stated “The study participants were prisoners at Gondar city who stayed for four and above (serving a1sentence or awaiting sentence) and whose age is 18 and above”. In Ethiopia is there any prisoners whose age are below 18?

2. In sample size calculation the authors stated that “The required sample size to address

the objectives of this study was calculated by the 1:10 ratio”. Since the authors used rule of thumb method to calculate the sample size which is very conservative and heavily affect the power of the study. For SEM, to apply the above method the data should be multivariate normally distributed and the authors actually not consider these assumption while determining the required sample size. Obviously, most of the variable included in the study have ordinal natures, my concern is how the author mediated these controversial ideas?

I strongly suggest the authors to use other powerful statistical methods to calculated the required sample size to answer the given research questions rather than using conservative method.

3. During the sample selection the authors stated “A simple random sampling technique was used to select participants”, the authors kindly suggested to clearly describe how simple random sampling was employed in the study population?

4. The authors stated “Data related to socio-demographic and clinical factors were collected by using semi-structure and pre-tested questionnaire.” Where and when the questionary was pretested?

5. The authors used WHOQoL-BREF tools to assess the Health related quality of life the prisoners. However, the tool basically developed and validated for non-health population. Further more the tool is developed to assess the Health related quality of life patients of acute disease in two weeks and for chronic patient in one month. There for the tool is not relevant for the these study population. Therefore, the authors kindly explain why used these tool for such population

**4: Statistical analysis**

1. The authors suggested the analysis was done using STATA and AMOS software. What is the important of analyzing the data using two software’s?

2. The authors suggested that “In this study, to reduce the number of parameters in the analysis and maintain a reasonable degree of freedom for the model”, why the authors want to reduce the parameters? As long as the required sample size was used why not including all parameters stayed in the model?

**3: Result**

1. In the result section of the manuscript, the authors stated that “The correlation analysis of all the domains of HRQoL and the global HRQoL done using spearman’s rank correlation coefficient.” However, the Overall HRQoL, and its four domains are obtained by taking the average of the corresponding indicator variables and these leads to the overall HRQoL and its domains have a continuous nature. My question is why you compute spearman’s rank correlation coefficient to assess the correlation between the continues variables?

Furthermore the authors does not mention these results in the method part of the manuscript. Your results should be follow the methodology of your study.

2. The authors also stated that “The final model was relatively simplify and best fitted model.” You mean that your model is correctly estimate parameters of the model?

3. The authors stated that “The unstandardized regression coefficients in table 5 represented the amount of change in the……”. If you used unstandardized regression coefficients you are not allowed to compare the coefficients with each other’s, to do so you have to report standardized regression coefficients .

4. The authors also reports direct, indirect, and total effect of the variables, during interpreting the total effect of the variables on the outcome variables, the two variables (the one have direct effect t and indirect effect through mediating variable then have total effect) may not have the same unit of measurement. Therefore how to interpret the total effect for such scenarios?

5. Finally, the way of presentation of the result is not good, the table the author used is not readable, to increase the quality of the manuscript clearly present your results.

4: **discussion**

1. In the recommendation part of the manuscript some of the statements are written multiple times therefore the authors suggested to avoid writing sentences having the same idea over the manuscript.

2. Generally, the way you discuss the significant variable was completely not good. The justification the author give for the significant variable was very raw. Furthermore as I said in measurement part of the manuscript, most of your explanation were not related to what you measured(different from the tool). These makes you result unreliable and hard to comment on it.

**5: Conclusion**

Generally the study was done in single site the author could not have to make generalize for the whole population**.**
